# Protocol for evaluating protein intake-induced feeding inhibition in *Drosophila* using a pre-feeding approach

**DOI:** 10.1016/j.xpro.2025.104056

**Published:** 2025-09-03

**Authors:** Yinuo Wang, Xiaotong Wang, Houqi Tan, Kaifeng Xiao, Yan Zhu, Yan Li, Yang Yang

**Affiliations:** 1 Institute of Biophysics, State Key Laboratory of Cognitive Science and Mental Health, Chinese Academy of Sciences, Beijing 100101, China; 2 University of Chinese Academy of Sciences, Beijing 100049, China; 3 Sino-Danish Center for Education and Research, Beijing 100190, China

**Keywords:** Genetics, Model Organisms, Neuroscience, Behavior

## Abstract

Consumption of dietary protein has been shown to elicit the strongest satiety among macronutrients. Protein intake-induced feeding inhibition (PIFI) plays a critical role in the maintenance of nutrition homeostasis. Here, we present an optogenetic pre-feeding protocol for studying the neural mechanisms underlying PIFI in *Drosophila*. We describe steps for preparing and loading flies into feeding chambers, quantifying food consumption, estimating excretion levels following the feeding test, and evaluating the inhibitory effect of dietary protein.

For complete details on the use and execution of this protocol, please refer to Li et al. and Sun et al.^1^^,^^2^

## Highlights

• Guidance on the pre-feeding assay for evaluating feeding inhibition in *Drosophila*
• Steps for optogenetic manipulation of neural activity in specific feeding stages
• Procedures for assessing natural feeding behavior with no anesthesia of the animal

## Abstract (editor highlights)

Publisher’s note: Undertaking any experimental protocol requires adherence to local institutional guidelines for laboratory safety and ethics.

## Graphical abstract

**Figure:**
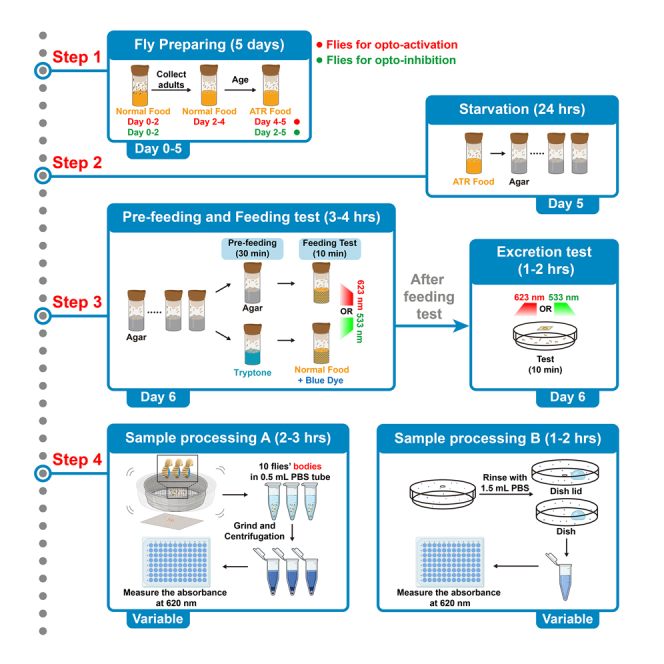

## Before you begin

### Innovation

Feeding is essential for the maintenance of nutrient homeostasis. Excessive protein intake, in particular, leads to metabolic imbalances and impairs normal growth and development. In *Drosophila*, we previously reported the feeding regulation termed protein intake-induced feeding inhibition (PIFI),^2^ characterized by a rapid reduction in food intake within 10 min following a 30-min protein consumption. However, neural circuits underlying PIFI regulation remain poorly understood.

To evaluate PIFI, we established the pre-feeding assay^2^ composed of three feeding stages, including starvation, pre-feeding, and feeding test. To precisely manipulate candidate neurons that participate in PIFI regulation, we utilize the optogenetic approach, which enables precise and timely activation and/or inhibition of neurons in specific feeding stages. In addition, an excretion test is supplemented following the feeding test to avoid confusing the effects from these two regulations.

In this protocol, the 10-min feeding test is performed immediately after the pre-feeding step. Thus, methods using individual flies and/or liquid food^3^^,^^4^^,^^5^^,^^6^ are not applicable. To obtain natural feeding conditions, we utilize the corn meal food and fly bottles that are used in cultivation for feeding test. Moreover, feeding bottles are warmed up with wild-type flies to provide a safe and comfortable environment. In addition, fruit flies are gently transferred without anesthesia. These settings allow animals to display feeding behavior without any interference.

In conclusion, we describe an optogenetic pre-feeding protocol for rapid assessment of PIFI and support the functional dissection of key neurons underlying PIFI in *Drosophila*.

### Prepare tools

1. Prepare an optogenetic unit for providing a light stimulus (Figure 1A).
  a. Wrap the light strip inside a container with the size of approx. 100 mm in diameter and 100 mm in height, allowing for one fly bottle to fit in. For opto-activation, use a 623 nm-wavelength light; for opto-inhibition, use a 533 nm-wavelength light.
  b. Connect the light strip to the power supply.
  c. Turn on the power, check the light intensity in the middle of the container by using Digital Optical Laser Power Meter, and adjust the intensity to 0.79 mW/mm^2^ (623 nm light) and 0.03 mW/mm^2^ (533 nm light) according to previous studies.^7^^,^^8^

**Figure 1:**
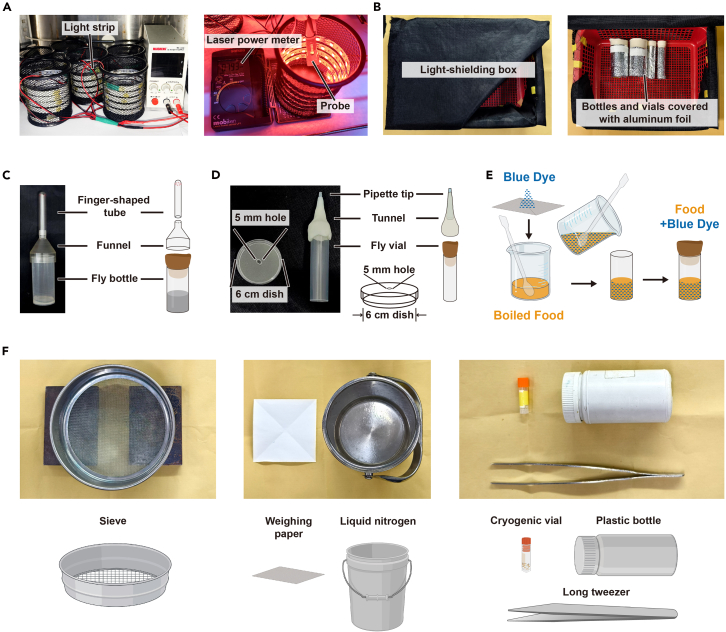
Tools and optogenetic units used in the feeding test and excretion test (A) The optogenetic unit demonstrates the components utilized for light stimulation, and the measurement section quantifies light intensity in 0.79 mW/mm^2^ (623 nm light) and 0.03 mW/mm^2^ (533 nm light). (B) The box is wrapped in black cloth (left). The fly bottles and fly vials are covered with aluminum foil and are put in the box (right). (C) Schematic diagram of the dispenser for splitting fly groups, including a finger-shaped tube, a funnel and a fly bottle. (D) Schematic diagram of the excretion test unit including a 6 cm dish with a 5 mm hole, and the transfer tunnel including a fly vial and a tunnel with a cut pipette tip. (E) Preparation of feeding test food by adding the brilliant blue dye to the Normal Food at 65°C–90°C. (F) Schematic diagram of the tools required during sample processing, including sieve (sieve aperture size, 710 μm), weighing paper, liquid nitrogen, cryogenic vial, plastic bottle, and long tweezer.
2. Prepare several aluminum foil sleeves for fly bottles and fly vials and a box wrapped with black cloth to prevent light exposure during fly cultivation (Figure 1B).
3. Prepare a dispenser for fly groups to be split into approx. 100 (Figure 1C).
  a. Prepare a funnel with an inner diameter slightly larger than the fly bottle’s outer diameter. Prepare a finger-shaped tube with an inner diameter slightly larger than the funnel’s thinner end. Ensure that the funnel smoothly connects to both the fly bottle and the finger-shaped tube.
  b. Count 100 anesthetized flies and transfer them into the finger-shaped tube. Draw a line to label the volume of the 100 flies, and use this line as a reference to split fly groups of 100 for all experiments. Prepare two labeled finger-shaped tubes for rotational use.
  c. Alternatively, draw a reference line on the finger-shaped tube for approx. 30 flies.
4. Prepare the excretion test unit (Figure 1D).
  a. Prepare 6 cm dishes to be used as excretion dishes. Poke a small hole of approx. 5 mm in diameter into the center of the dish lid.
  b. Cut the tip (approx. 3 mm) of a 1 mL blue pipette tip to fit through the small hole on the lid. Ensure that flies can easily pass the open end of pipette tip into the excretion dish unhindered.
  c. Prepare a tunnel with an inner diameter slightly larger than the outer diameter of the fly vial.
  d. Connect the pipette tip to the thinner end of the tunnel and use the Parafilm to fasten pipette tip to tunnel. Use this apparatus to transfer flies into individual dishes without the need for anesthesia.

### Prepare the normal food for fly cultivation

**Timing: 6 h**

5. Food cooking according to the food recipe in materials and equipment.
  a. For 1 L of Normal Food, weigh out 68 g cornmeal, 16 g yeast, 9 g soybean powder, and 5 g agar.
  b. Add 1.0 L distilled water into an electric cooker, and then transfer weighed compounds above into the cooker. Turn on the power and stir continuously until boiling.
  c. Maintain the mix at boiling level for 30 min.
  d. Switch to KEEP-WARM mode and allow the food to slowly cool down to 75°C. Add 0.726 g calcium chloride (anhydrous) and 43 g maltose. Stir continuously for 2–3 min, until completely resolved.
  e. Wait until the mix has cooled down to 65°C, then add 5.0 mL propionic acid and stir continuously for 2–3 min until fully mixed.
6. Pour the hot Normal Food into fly bottles.
  a. Pour approx. 20 mL into each bottle. If ATR was added, label the bottles as ATR food (indicate final concentration).
  b. Cover the food bottle with a piece of sterilized double-layer gauze;
  c. Leave the food solidified under a clean and ventilated environment for about 3 h until no obvious steam on the wall of the fly bottle.

***Note:*** Ensure no fly or other insects getting into the bottles.

7. Store the Normal Food at 4°C (refrigerator or cold room) for max. 1 week.

### Prepare ATR food for optogenetic experiments

***Note:*** All-Trans-Retina (ATR) is light-sensitive! It therefore should only be added to the food 1–2 days prior to conducting the optogenetic experiments (ATR food). Thus, the entire process of handling the ATR should be conducted under dark conditions, with minimal exposure to light.

8. Prepare a 200 mM ATR stock solution by dissolving 1.0 g of ATR powder in 17.578 mL anhydrous ethanol. Vortex until completely dissolved.
9. (Example for 100 mL) Aliquot 100 mL of the Normal Food into 500 mL beakers. Add the ATR solution to the food at 60°C–65°C. For opto-activation experiments, add 200 μL ATR stock solution into 100 mL Normal Food (final concentration 0.4 mM).^9^ For opto-inhibition experiments, add 500 μL ATR stock solution into 100 mL Normal Food (final concentration 1 mM).^8^^,^^10^
10. Stir the mix thoroughly using a medicine spoon.
11. The food is poured and stored in the same way as described for the Normal Food (described in steps 6 and 7 of “prepare the normal food for fly cultivation”).

### Prepare fly food used for experiments

**Timing: 1 h**

12. 1% agar food for starvation treatment (Example for 100 mL)
  a. Add 1.0 g agar to the 100 mL distilled water and stir until thoroughly mixed.
  b. Heat the agar mixture in the microwave oven until boiling.
  c. Move the mix out from the microwave and stir. Repeat the boiling-stirring procedure three times to ensure the agar is completely dissolved.
13. 1.7% tryptone food^2^ for pre-feeding treatment (Example for 100 mL).
  a. Add 1.7 g tryptone to 100 mL of freshly boiled 1% agar mix.
  b. Stir continuously until the mixture is completely dissolved.
14. Feeding test food (Example for 100 mL) (Figure 1E)
  ***Note:*** Handle with care and avoid air drafts, as the dye easily disperses.
  a. Tryptone food used as test food.
    Gently add 0.5 g brilliant blue dye to 100 mL 1.7% tryptone food at 65°C. Stir thoroughly to ensure tryptone is completely dissolved.
  b. Normal Food used as test food.
    Gently add 1.0 g brilliant blue dye to 100 mL Normal Food at 65°C. Stir thoroughly until the mixture is fully dissolved.
15. The food is poured into fly bottles or vials, and stored in the same way of Normal Food (described in steps 6 and 7 of “prepare the normal food for fly cultivation”).

***Note:*** Food containers used in the light stimulation step should exhibit max light transparency, to allow for good light penetration. Ideally, use 100 mL glass beakers instead of fly bottles.

## Key resources table

**Table:** 

REAGENT or RESOURCE	SOURCE	IDENTIFIER
**Chemicals, peptides, and recombinant proteins**		

Agar	Solarbio	A8190
Cornmeal	Beijing Zhongchang Huahui Supermarket Co., Ltd.	N/A
Yeast	Hebeiruiqi Biotechnology Co., Ltd.	N/A
Maltose	Boao Biotechnology Co., Ltd.	N/A
Calcium chloride anhydrous	Beijing Yili Fine Chemicals Co., Ltd.	CAS#10043-52-4
Propionic acid	Sinopharm Chemical Reagent Co., Ltd.	CAS#79-09-4
Soybean powder	Beijing Zhongchang Huahui Supermarket Co., Ltd.	N/A
Tryptone (w/v 1.7%)	OXOID	LP0042
Brilliant Blue	Care	N/A
All-Trans-Retina (ATR)	Sigma	R2500
PBS	Sangon Biotech	B548117

**Experimental models: Organisms/strains**		

R67D01-p65AD	Li et al.^1^	N/A
TH-C-Gal4DBD	Xie et al.^11^	N/A
UAS-GtACR2	Bloomington *Drosophila* Stock Center (BDSC)	92987
UAS-CsChrimson	Bloomington *Drosophila* Stock Center (BDSC)	55135
*w*^*1118*^	Bloomington *Drosophila* Stock Center (BDSC)	5905

**Software and algorithms**		

GraphPad Prism 8	GraphPad Software	https://www.graphpad.com
Excel	Microsoft	https://www.microsoft.com/en-us/microsoft-365/excel
BioRender	BioRender	https://biorender.com

**Other**		

Eppendorf tube	Axygen	MCT-150-CLD
Absorbance plate reader-Infinite F50 Plus	Tecan	https://lifesciences.tecan.com/products/microplate_readers/infinite_f50
96-well cell culture plate	Costar	3599
Cryogenic vials	Biosharp	BS-20-ST
Finger-shaped tube (Ø×H: 18 mm × 95 mm)	Duowen Medical Equipment Store	N/A
Fly vial (Ø×H: 23 mm × 95 mm)	Haimen Jinning Experimental Equipment Business Department	N/A
Fly bottle (Ø×H: 50 mm × 100 mm)	Haimen Haojie Experimental Equipment Business Department	N/A
Automatic sample fast grinder	Shanghai Jingxin	Tissuelyser-24
Steel beads (Ø: 3 mm)	Guwanji Hardware Technology Co., Ltd	N/A
Sieve (Ø: 200 mm; sieve aperture size: 710 μm)	Xinxiang Kangda New Machinery Co., Ltd	Ø200∗25
Glass beaker (150 mL)	BOMEX	N/A
LED strip (623 nm) (L×W: 110 cm×10 mm)	Shenzhen Pengxing Optoelectronics Technology Co., Ltd	5050
LED strip (533 nm) (L×W: 110 cm×10 mm)	Shenzhen Pengxing Optoelectronics Technology Co., Ltd	5050
Digital optical laser power meter	SANWA	LP1
Power supply	MAISHENG	MN-305D
Electric cooker	PESKOE	CFXB230-300
Microwave oven	Panasonic	NN-SM319H

## Materials and equipment

**Table:** Normal food

Reagent	Final concentration (w/v)	Amount
Cornmeal	6.8%	68 g
Yeast	1.6%	16 g
Soybean powder	0.9%	9 g
Agar	0.5%	5 g
Calcium chloride (anhydrous)	0.0726%	0.726 g
Maltose	4.3%	43 g
Filtered water	N/A	1 L
**Total**	**N/A**	**1 L**

Store the Normal Food at 4°C (refrigerator or cold room) for max. 1 week.

## Step-by-step method details

### Fly crossing and cultivation

**Timing: 15–18 days**

This section describes how to cross and collect the flies for the experiments.

1. Select the fly driver strains (e.g. Gal4 or LexA) expressing in the desired neurons or cells. Cross these strains with flies carrying the optogenetic elements (e.g. CsChrimson or GtACR).
2. To set the experiments in a double-blind manner, label each bottle with a code to each bottle by a person other than the experimenter to ensure unbiased.
3. Rear flies using Normal Food, under standard conditions: 25°C, 40%–60% humidity, 12 h/12 h light/dark cycle.
4. Collect the progeny flies.
  a. When progeny flies start to eclose, remove all eclosed flies from the bottle at the end of the day.
  b. Collect the progeny flies every two days.
  c. Mark the collection day on the fly bottles and record this day as **Day 2.**
5. Transfer flies into the ATR-containing Normal Food and age to **Day 5.**
  a. For the opto-inhibition experiment, flies are transferred to 1 mM ATR food on **Day 2.**
  b. For the opto-activation experiment, flies are aged for 2 days in the Normal Food and transferred to 0.4 mM ATR food on **Day 4.**

***Note:*** Wrap the bottles with aluminum foil sleeves and place them inside the box wrapped with black cloth. Keep the box in a completely dark environment to prevent light exposure.

***Note:*** Avoid CO_2_ anesthesia of the flies. If unavoidable, allow flies to recover for at least 24 h before proceeding with the following steps.

### Starvation treatment

**Timing: 1 day**

This section describes the details of starvation treatment of the flies.

***Note:*** This step should be conducted in a dark environment. Fly bottles are wrapped with aluminum foil sleeves and put into box wrapped with black cloth.

6. Gently transfer all the collected flies with the same double-blind code into the same agar bottle.
7. Split the flies into groups of approximately 100 flies using the fly dispenser (described in prepare tools). Avoid any anesthesia.
  a. Transfer each group into a new agar bottle clearly labeled with the above-mentioned double-blind code.
  b. Repeat this process until all the flies are divided into groups of 100 per bottle (Figure 2A).

**Figure 2:**
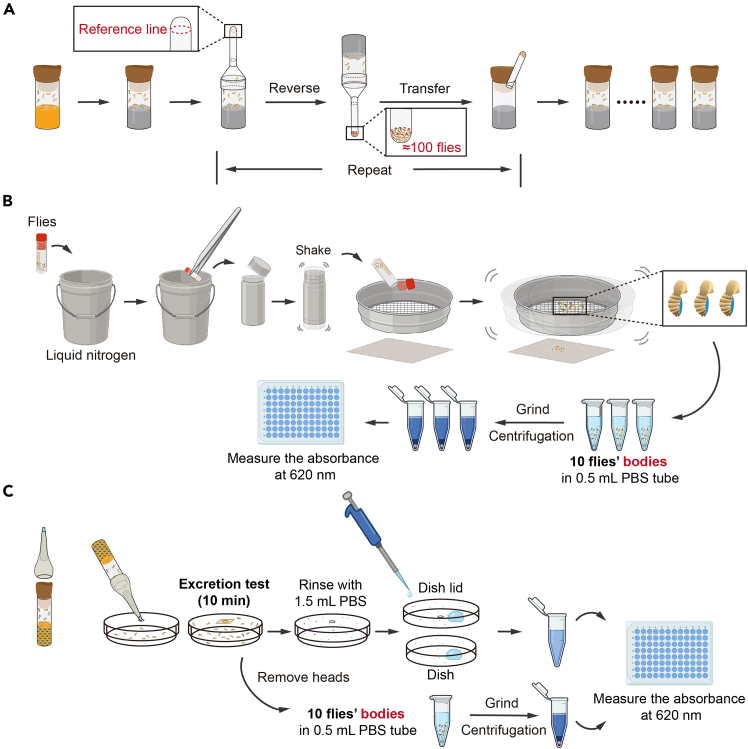
Steps for starvation and sample processing (A) Procedure of splitting flies into groups of approximately 100 flies per bottle. (B) Procedure of processing the fly samples. Remove the heads, legs, and wings. Grind the bodies and measure the absorbance of residual dye in the abdomen. (C) Procedure of sample processing for excretion test. Rinse the dish with PBS and measure the absorbance of the solution. Fly samples are processed according to (B). See also Methods video S1.
  c. Alternatively, flies can be divided into groups of 30 and transferred into fly vials.
8. Starve the flies for 24 h in a 25°C-facility, at a humidity of 40%–60%.

### Warm-up of the food bottles for behavioral experiments

**Timing: 4 h**

This section describes how to warm-up food bottles used in behavioral experiments. Alternatively, warm-up fly vials when small-size groups of flies are used.

***Note:*** The time periods of both pre-feeding and feeding test are short, so it is critical to provide a natural and comfortable environment for flies to limit the adaptation time. This step makes the food bottles saturated with familiar odors of flies, which allows flies to display feeding behavior under natural conditions. This step is important for obtaining accurate and consistent results.

9. Remove the food bottles from the refrigerator and allow them to reach room temperature, for a minimum of 3 h.
10. Place approx. 50–100 unanesthetized wild-type flies into each bottle. Allow the flies to stay in the bottles for 30 min.
11. Carefully remove all flies from the bottles, and double-check that all flies have been removed.

### Pre-feeding treatment

**Timing: 30 min**

This section describes the details of pre-feeding treatment of the flies in the pre-feeding assay. Alternatively, use fly vials when small-size groups of flies are used.

12. Gently transfer the starved flies into the pre-feeding bottles with 1.7% tryptone or 1% agar without blue dye.
13. Limit the space between food and flies by pressing the bottle stopper down to approx. 2 cm above the food surface, allowing flies to be in contact with the food more often.
14. Place the bottles into a dark environment and adjust to 25°C, 40%–60% humidity. Allow flies to consume their food undisturbed for 30 min. Avoid any vibration or noise.

***Note:*** Set a timer to ensure a strict pre-feeding time of exactly 30 min.

### Feeding test

**Timing: 10 min**

This section describes how to perform feeding experiments with the optogenetic manipulation. Alternatively, use fly vials when small-size groups of flies are used.

15. Gently transfer the pre-fed flies into the test bottles containing Normal Food with blue dye.
16. Press the bottle stopper down to approx. 2 cm above the food surface.
17. Place one test bottle in one optogenetic unit.
  a. For opto-activation, use the units with the 623 nm-wavelength light source.
  b. For opto-inhibition, use the units with 533 nm-wavelength light source. For the light-OFF control groups, ensure the light remains OFF.
18. Place the bottles under 25°C and 40%–60% humidity. Allow flies to consume food for 10 min undisturbed. Avoid any vibration or noise.

***Note:*** Set a timer to ensure a strict feeding timing of exactly 10 min.

19. Upon completing the feeding time, take a close observation on the wall of the test bottle to check whether there are excretion dots.
  a. If there are blue excretion dots observed, transfer flies into the empty fly vials clearly labelled with the double-blind code and the feeding conditions.
  b. Place the fly vials and the test bottle into a −80°C freezer for at least 2 h. The collected samples can be preserved for up to two weeks at this temperature.
20. If no blue dots are observed, transfer flies into the excretion dishes with clear labels and subject them for the excretion test in the next section.

***Note:*** If approx. 100 flies are tested in fly bottle, use the finger-shaped tube to gently split the flies into three dishes with approx. 30 per dish.

***Note:*** To examine the protein consumption levels of the hungry flies, skip the pre-feeding steps (steps 12–14). Instead, transfer the starved flies straight into the 1.7% tryptone food containing blue dye as described in step 15 *(*step 11 *->* step 15).

### Excretion test

**Timing: 10 min**

This section describes how to test the excretion levels after food consumption.

21. Following the feeding test, gently transfer the flies into the excretion dishes through the hole on the lid (described in section prepare tools) and seal the hole with a piece of tape.
22. Place the excretion dishes in the optogenetic units. Set the light conditions according to step 17*.*
23. Allow the flies to excrete undisturbed for 10 min. Avoid any vibration or noise.
24. Freeze the flies in the excretion dishes in the −80°C freezer.

### Sample processing for feeding test

**Timing: 2–3 h**

This section is to quantify the amount of food consumption of flies (Figure 2B) (Methods video S1).

25. Collect all the female and male flies into two 2 mL cryogenic vials.
  a. Remove the vials containing the fly samples from the −80°C freezer (following step 24).
  b. Pour the flies of one fly vial onto a piece of weighing paper.
  c. Separately collect all the female and male flies into two 2 mL cryogenic vials with the double-blind code, ensuring that the code is correct.
26. Remove the flies’ heads, legs, and wings using tools depicted in Figure 1F.
  a. Slightly loosen the cap of the cryogenic vials and submerge them into the liquid nitrogen tank.
  b. Wait until bubbling ceases, pick up the cryogenic vials with a long tweezer. Put them into an empty plastic bottle, and then tighten the lid.
  c. Shake the bottle vigorously 7–8 times to detach the heads, legs, and wings from all fly bodies.
    ***Note:*** Practice and find a proper force of shaking to ensure the head detach, the chest and abdomen remain intact.
  d. Pour the flies onto a sieve prepared prior to this procedure. The smaller parts, including heads, legs and wings, should pass through the sieve, while the fly bodies should be retained in the sieve.
  e. Transfer the fly bodies onto a piece of weighing paper with the appropriate labels.
27. Add one single steel bead and 500 μL PBS solution into each Eppendorf tube serving as the grind tubes.
28. Divide the flies into grind tubes clearly labelled with the double-blind codes and the feeding groups.
  a. For large group flies (approx. 100), divide flies into three groups of 10 and transfer into three grind tubes.
  b. For small-size group flies (approx. 30), collect 10 flies into one grind tube.
29. Grind the dye-ingested flies.
  a. Grind the flies in the grind tubes using an automatic grinder at 60 Hz for 10 s. Ensure all flies are fragmentized.
  b. Centrifuge the tubes at 15000 × *g* for 30 min.
30. Carefully pipette supernatant from each grind tube and load into a 96-well plate. PBS is used for the blank control by loading three wells.
  a. For large group flies (approx. 100), pipette 100 μL supernatant from each grind tube and load into one well of a 96-well plate.
  b. For small-size group flies (approx. 30), pipette 100 μL supernatant three times from each grind tube as the loading control.

***Note:*** The pellets will float easily, thus handling the tubes carefully is critical to avoid the resuspension of the pellets. When pipetting the supernatant, avoid disturbing or pipetting the upper fat layer present on top of the pellet.

31. Measure the absorbance at 620 nm using an absorbance plate reader, and record using the correct labels.

Methods video S1. Sample processing, related to steps 26–30 and Figure 2B Remove the heads, legs and wings, and grind the samples.

### Sample processing for excretion test

This section is to quantify the amount of fly excretion (Figure 2C).

32. For test bottles, wash the blue excretion dots with PBS and measure the excretion levels according to the previous study.^12^
33. For excretion dishes, process the fly samples and excretion dishes separately.
  a. Take the excretion dishes with fly samples out of the −80°C freezer.
  b. Pour the flies onto a piece of weighing paper and count the number of flies (N_flies_). Collect all the flies into a 2 mL cryogenic vial.
  c. Process all fly samples according to steps 25–31*.*
  d. Rinse each dish repeatedly with 1.5 mL PBS until all the visible blue excretion dots are dissolved in the PBS solution.
34. Pipette 100 μL rinse liquid into one well of a 96-well plate, and repeat three times for each excretion dish.
35. Measure the absorbance at 620 nm using an absorbance plate reader.

### Data analysis

This section describes statistical analysis of PIFI effect and excretion level of flies.

36. Following sample processing, export the data recorded by the absorbance plate reader.
37. Average the reading results of the 3 loading repeats of PBS as “Average PBS”.
38. To measure Food Consumption (FC), average the readings of the 3 repeats from the same cryogenic vial of agar or tryptone pre-feeding groups and subtract the Average PBS, recorded as “FC_Agar(i)_” or “FC_Tryptone(i)_”.

FC_Agar(i)_ = (Repeat 1+Repeat 2+Repeat 3)/3–Average PBS

FC_Tryptone(i)_ = (Repeat 1+Repeat 2+Repeat 3)/3–Average PBS

39. To quantify the food consumption of n trails of PIFI experiments, average the FC_Agar(i)_ of the maternal control groups as “Average (FC_Maternal Control_)”.

$$\text{Average}\left({\text{FC}}_{\text{Maternal}\;\text{Control}}\right)=\frac{\left(\sum_{i=1}^{n}{\text{FC}}_{\text{Agar}\left(i\right)}\right)}{n}$$

40. To obtain the Relative Food Consumption (RFC), normalize each group to the maternal control.

$${\text{RFC}}_{\text{Agar}\left(i\right)}=\frac{{\text{FC}}_{\text{Agar}\left(i\right)}}{\text{Average}\left({\text{FC}}_{\text{Maternal}\;\text{Control}}\right)}\;i=1\ldots n$$

$${\text{RFC}}_{\text{Tryptone}\left(i\right)}=\frac{{\text{FC}}_{\text{Tryptone}\left(i\right)}}{\text{Average}\left({\text{FC}}_{\text{Maternal}\;\text{Control}}\right)}\;i=1\ldots n$$

41. To obtain the Suppression Index (SI) for each genotype, calculate the Average(RFC_Agar_) and subtract it from the RFC_Tryptone(i)_. The difference is divided by Average(RFC_Agar_), and the ratio is marked as the Suppression Index of Tryptone (SI_T_).

$$\text{Average}\left({\text{RFC}}_{A\text{gar}}\right)=\frac{\left(\sum_{i=1}^{n}{\text{RFC}}_{\text{Agar}\left(i\right)}\right)}{n}$$

$${\text{SI}}_{T\left(i\right)}=\frac{{\text{RFC}}_{\text{Tryptone}\left(i\right)}-\text{Average}\left({\text{RFC}}_{\text{Agar}}\right)}{\text{Average}\left({\text{RFC}}_{\text{Agar}}\right)}\;i=1\ldots n$$

42. To obtain RFC for the tryptone feeding test, average the FC_Tryptone(i)_ of the maternal control groups as “Average(FC_Maternal Control_)”.

$$\text{Average}\left({\text{FC}}_{\text{Maternal}\;\text{Control}}\right)=\frac{\left(\sum_{i=1}^{n}{\text{FC}}_{\text{Tryptone}\left(i\right)}\right)}{n}$$

$${\text{RFC}}_{\text{Tryptone}\left(i\right)}=\frac{{\text{FC}}_{\text{Tryptone}\left(i\right)}}{\text{Average}\left({\text{FC}}_{\text{Maternal}\;\text{Control}}\right)}\;i=1\ldots n$$

43. To obtain Relative Excretion (REX) for the Excretion test, adjust the value by dividing N_flies_ and multiplying by 30 for each repeat. Calculate the excretion level as Excretion_Agar(i)_ or Excretion_Tryptone(i)_ following the steps described in steps 37 and 38*.* Normalize each group to the food consumption of maternal control.

$${\text{REX}}_{\text{Agar}\left(i\right)}=\frac{{\text{Excretion}}_{\text{Agar}\left(i\right)}}{\text{Average}\left({\text{FC}}_{\text{Maternal}\;\text{Control}}\right)}\;i=1\ldots n$$

$${\text{REX}}_{\text{Tryptone}\left(i\right)}=\frac{{\text{Excretion}}_{\text{Tryptone}\left(i\right)}}{\text{Average}\left({\text{FC}}_{\text{Maternal}\;\text{Control}}\right)}\;i=1\ldots n$$

44. Calculate the Excretion-to-Retention Ratio (ERR) by dividing Excretion_Agar(i)_ and Excretion_Tryptone(i)_ with the averaged FC_Agar_ and FC_Tryptone_, respectively.

$${\text{ERR}}_{A\text{gar}\left(i\right)}=\frac{{\text{Excretion}}_{A\text{gar}\left(i\right)}}{\text{Average}\left({\text{FC}}_{\text{Agar}}\right)}\;i=1\ldots n$$

$${\text{ERR}}_{T\text{ryptone}\left(i\right)}=\frac{{\text{Excretion}}_{T\text{ryptone}\left(i\right)}}{\text{Average}\left({\text{FC}}_{T\text{ryptone}}\right)}\;i=1\ldots n$$

45. Analyze all data using GraphPad Prism and plot as mean ± standard error of mean (S.E.M.). Use Student’s *t* test to compare two groups, and One-way ANOVA followed by Dunnett’s multiple comparisons test for comparing among groups. Use Two-way ANOVA to determine the interaction between two factors. ∗, *p* < 0.05; ∗∗, *p* < 0.01; ∗∗∗, *p* < 0.001. See also Table S2.

## Expected outcomes

Here, we present a reliable and efficient method for investigating the neural mechanisms underlying the inhibitory effects of protein satiety on food consumption in fruit flies, named protein intake-induced feeding inhibition (PIFI) assay.^2^ Flies are pre-fed with Agar or Tryptone, and the Relative Food Consumption (RFC) is measured with the Normal Food with blue dye. The Suppression Index (SI_T_) is calculated to evaluate the inhibitory effect of protein-intake during the pre-feeding period on the following food consumption during the test period. The optogenetic approach is utilized to timely activate or inhibit a certain group of neurons during the test period of PIFI assay. Using this protocol, it is possible to determine whether these neurons are required for PIFI regulation.

**Figure 3:**
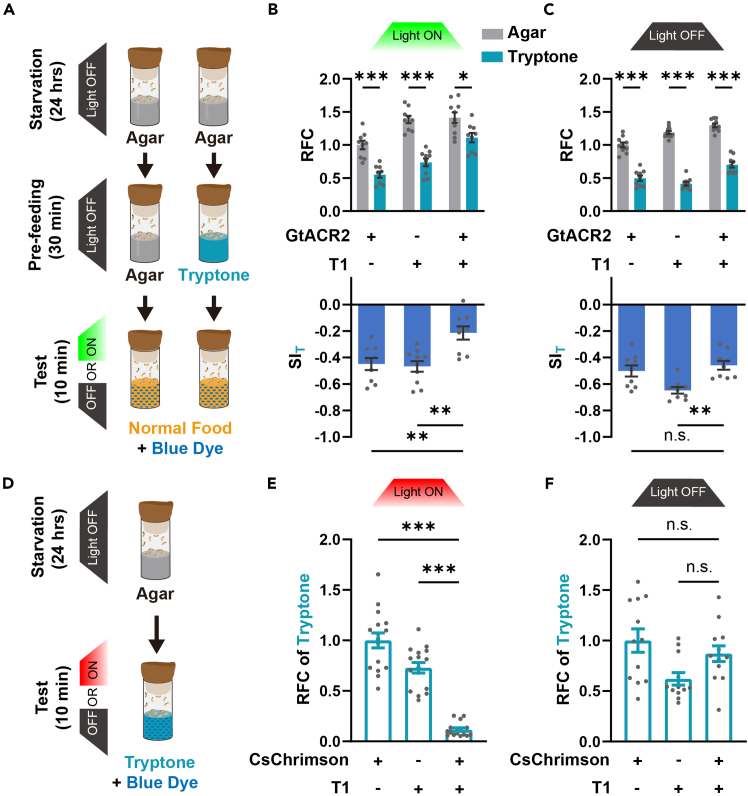
Behavior test for pre-feeding test and protein consumption test under optogenetic conditions (A) Schematic diagram of optogenetic manipulation in the pre-feeding test. (B and C) Optogenetic silencing of T1-DANs inhibited PIFI (B), but not in the light OFF control group (C). *n* = 9–11. (D) Diagram illustrating optogenetic manipulation applied in the tryptone consumption test. (E and F) Opto-activation of T1-DANs inhibited tryptone feeding (E), but not in the light OFF control group (F). *n*=12–16. *n* represents the number of trials. Student’s t test for Relative Food Consumption (RFC) in (B) and (C). One-way ANOVA, Dunnett test for Relative Food Consumption (RFC) in (E)–(F) and for Suppression Index of Tryptone (SI_T_) in (B) and (C). ∗*p* < 0.05. ∗∗*p* < 0.01. ∗∗∗*p* < 0.001. n.s. indicates no statistical significance. The data are shown in mean ± S.E.M. See also Tables S1 and S2.

A pair of dopaminergic neurons, namely T1-DANs, have been previously reported to play a crucial role in PIFI.^1^ Here, we demonstrate how to study the function of candidate neurons in regulating PIFI effect by using these neurons. When we opto-inhibited T1-DANs during the test period (Figure 3A), we found that the SI_T_ was significantly reduced in the experimental group compared to the parental control (Figure 3B). However, no such decrease was observed in the light-OFF control group (Figure 3C). In comparison, while opto-activating T1-DANs (Figure 3D) significantly suppressed protein intake in hungry flies (Figure 3E), these flies displayed similar food intake when the light was OFF (Figure 3F). These results indicate that T1-DANs represent a protein satiety signal, which is required for PIFI effects and which is sufficient to inhibit protein consumption.

**Figure 4:**
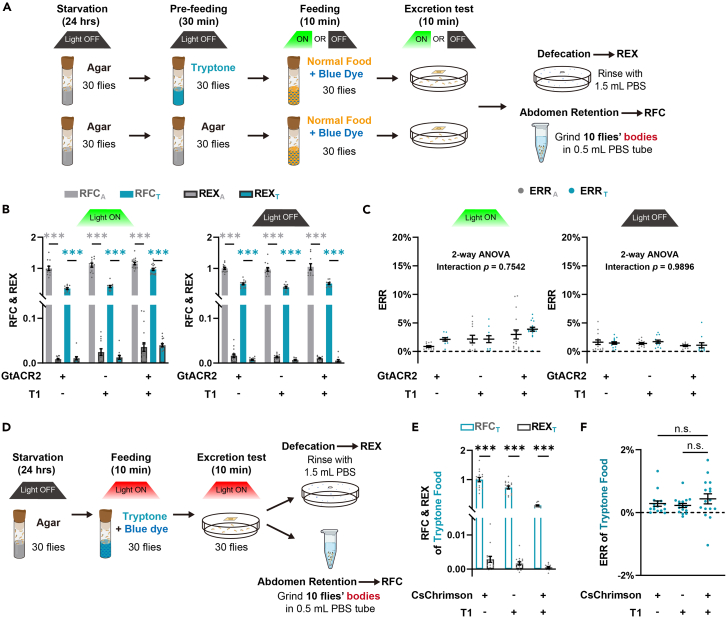
Excretion test under optogenetic conditions (A) Schematic diagram illustrating the excretion test after the pre-feeding test, with opto-inhibition of T1-DANs used as an example. (B) Compared to the food consumption, flies showed small amount excretion in all groups. *n*=10–17. (C) Excretion-to-Retention Ratio (ERR) were less than 5% and comparable among groups. *n*=10–17. (D) Schematic diagram of the excretion test after the protein consumption test, with opto-activation of T1-DANs as an example. (E) Compared to the food consumption, flies showed small amount excretion when the T1-DANs were activated or not. *n*=16. (F) The ERR showed no significant difference among groups. *n*=16. *n* represents the number of trials. Student’s t test for comparative analysis of relative food consumption and relative excretion levels in (B) and (E). Two-way ANOVA, Dunnett test for excretion to retention ratio in (C). One-way ANOVA, Dunnett test for excretion ratio in (F). ∗*p* < 0.05. ∗∗*p* < 0.01. ∗∗∗*p* < 0.001. n.s. indicates no statistical significance. The data are shown in mean ± S.E.M. See also Tables S1 and S2.

Notably, in this protocol, we evaluated the amount of food intake by measuring the absorbance of residual dye in fly abdomen. Nevertheless, in addition to food intake, excretion may also affect the amount of residual dye. Therefore, to address this issue, we adopted a method to examine the excretion levels of flies when manipulating candidate neurons.^12^ In our case, there was no excretion dot on the wall of the test bottles. To further verify whether the excretion was affected by opto-activating or inhibiting T1-DANs, we transferred the flies into the excretion dish for 10-min excretion test after the feeding test (Figure 4A). Compared to the food retained in flies (recorded as the relative food consumption, RFC), the Relative Excretion (REX) levels were low in both light-ON (opto-inhibiting T1 neurons) and light-OFF groups (Figures 4B and 4C). In addition, the Excretion to Retention Ratio (ERR) were less than 5% in all groups, and opto-inhibition of T1-DANs exhibited excretion levels comparable to genetic control groups under both light-ON and light-OFF conditions (Figures 4D and 4E). Similarly, the excretion levels were low (less than 1%) compared to food retained in flies when T1-DANs were opto-activated (Figure 4F). Together, these results indicated that T1-DANs are not involved in excretion regulation.

Taken together, this protocol enables researchers to evaluate protein intake-induced feeding inhibition and investigate the key neurons that are required for the regulation of PIFI through optogenetic manipulations. In addition, the method of our excretion test can also be used to determine whether the neurons regulate feeding or excretion.

## Limitations

In this PIFI assay, we used the Normal Food (the components are described in steps 12–15 of “prepare fly food used for experiments”) as test food, whose composition is complex, containing nutrients of protein, sugar, fat, et al. Therefore, SI_T_ may change if the proportion of different components changes. For more precise and stable results, researchers can use a specific proportion of selected nutrients such as sugar, protein, and fat as the test food. Notably, if the candidate neurons under investigation play a negative role in regulating PIFI, opto-activation should be used in the assay instead of opto-inhibition. When the function of the candidate neurons has not been determined, both inhibition and activation approaches should be tested.

In our assay, where the 10-min feeding test immediately follows the pre-feeding,^2^ the dye method^13^ reliably represents the feeding amount with minimal interference from excretion. However, if the test duration extends to >30 min, a considerable amount of the dye is excreted. As a consequence, the dye remained in the abdomen fails to accurately reflect actual food consumption because of the excretion that occurred during this time span. Therefore, to evaluate the PIFI effects across longer periods, different intervals need to be added between the pre-feeding and the 10-min feeding test. Alternatively, it may be feasible to implement a continues feeding test using methods such as FLIC,^6^ FlyPad^14^ and Expresso system.^5^

Our method described here is designed to measure food intake in large groups of flies under natural and undisturbed environments. To access feeding patterns of individual flies, methods such as dFRAME, ARC, Expresso system, FLIC, and FlyPad are recommended.^4^^,^^5^^,^^6^^,^^14^^,^^15^

## Troubleshooting

### Problem 1

The body size of flies varies or is too small (related to steps 1–4).

### Potential solution

Reduce the number of parental flies or the time of egg-laying. In general, 30 females and 15 males in each bottle and a two-day period of egg laying is recommended.

### Problem 2

Lethal or abnormal development of flies (related to step 4).

### Potential solution

• Use *tubulin*-Gal80^ts^ to restrict the expression of optogenetic elements during the development.
• Maintain the fruit flies in complete darkness to prevent leakage activation or inhibition of related neurons.

### Problem 3

Flies escaped during the transfer between bottles (related to steps 5, 6, 12, 15, and 21).

### Potential solution

Move gently during the transfer, and avoid repeated tapping of the bottles.

### Problem 4

The reading of the absorbance is below the reliable range (related to step 35).

### Potential solutions

• Slightly increase the concentration of dye added to the test food (step 14 in the section of “before you begin”).
• Prolong the time up to 2 h (step 10 in the section of “warm-up of the food bottles for behavioral experiments”).
• Strictly control the room temperature and humidity to 25±1°C and 50±10%. Inappropriate temperature and humidity may affect feeding and excretion (steps 14 and 18).
• Make sure that the transfer of flies is done gently and fast (steps 12, 15, and 21).
• Use freshly prepared food (less than 5 days) during each test. The food should be used no more than twice (steps 12–15 in the section of “before you begin”).
• Considering the difference in depth of neurons in the brain, and the transparency of test bottles, the different neurons require varying light intensity to be opto-activated or opto-inhibited. It is essential to adjust the light intensity to the proper levels.

## Resource availability

### Lead contact

Further information and requests for resources and reagents should be directed to and will be fulfilled by the lead contact, Yang Yang (yangyang@ibp.ac.cn).

### Technical contact

For technical specifics on executing the protocol, Dr. Yang Yang (yangyang@ibp.ac.cn) will provide support to ensure its correct implementation.

### Materials availability

This study did not generate new unique reagents and materials.

### Data and code availability

• All data are available from the lead author upon request.
• This article does not report original code.
• Any additional information required to reanalyze the data reported in this article is available from the lead contact upon request.

## Acknowledgments

We thank Bloomington *Drosophila* Stock Center for providing fly strains. We thank Dr. Chang Liu, Dr. Jinghan Sun, Dr. Xiaobing Bai, and Dr. Xiaoyu Li for helpful discussion and technical support. We thank Dr. Torsten Juelich for editing the manuscript. Some cartoon materials were adopted from BioRender.com for the graphs. This work was supported by the 10.13039/501100012166National Key R&D Program of China (2019YFA0802402), Space Application System of China Manned Space Program (YYWT-0801-EXP-13), and the 10.13039/100014717National Natural Science Foundation of China (grants 32211540388, 32061143011, and 31970947) to Y.L.

## Author contributions

Y.L. and Y.Y. designed the behavior assay. Y.L. secured funding. X.W., H.T., and K.X. performed the feeding behavioral experiments and analyzed the data. Y.Y. and Y.W. performed the excretion behavioral experiments and analyzed the data. H.T., K.X., and Y.W. prepared the figures. Y.Y. and X.W. wrote the manuscript. All authors read and revised the manuscript.

## Declaration of interests

The authors declare no competing interests.

## Supplemental information

Table S1. Fly genotypes in figures, related to Figures 3 and 4

Table S2. Statistical test used in experiments, related to step 45
